# Spatiotemporal patterns and clustering of prostate cancer incidence in China: a Bayesian modeling study of cancer registry data

**DOI:** 10.1186/s12889-026-26619-7

**Published:** 2026-02-14

**Authors:** Xu Zhu, Zhan Chen, Meng-Wei Ge, Attiq-Ur Rehman, Hong-Lin Chen, Hua Zhu, Bing Zheng

**Affiliations:** 1 Department of Urology, Affiliated Hospital 2 of Nantong University, Nantong, Jiangsu PR China; 2 Medical school, Nantong University, Nantong, Jiangsu PR China; 3 Institute of Urological Diseases, Nantong University, Affiliated Hospital 2 of Nantong University, Nantong, Jiangsu PR China; 4 School of Nursing and Rehabilitation, Nantong University, Nantong, Jiangsu PR China

**Keywords:** Prostate cancer, Spatial analysis, Bayesian spatiotemporal analysis, Cancer registry data, Spatiotemporal interaction model

## Abstract

**Purpose:**

Prostate cancer constitutes a major public health challenge in China; however, its spatiotemporal dynamics remain unclear. Clarifying these patterns is critical for guiding targeted prevention and control efforts to alleviate the disease burden.

**Materials and methods:**

A descriptive spatiotemporal study was conducted utilizing city-level prostate cancer registry data from 2013 to 2016. The data were retrieved from the Annual Reports on Cancer Registration in China 2016 to 2019 published by the Chinese National Cancer Center. The analytical framework integrated spatial autocorrelation analysis (global and local clustering) and Bayesian spatiotemporal modeling. Disease dynamics were comprehensively assessed using Bayesian spatiotemporal models, which incorporated structured and unstructured spatial effects, temporal trends, and spatiotemporal interactions.

**Results:**

Significant spatial clustering and geographic variation in prostate cancer incidence was identified. Rates were higher in southeast coastal regions and lower in northwest and northern China. Global Moran’s I values increased from 0.141 in 2013 to 0.173 in 2016 (all *P* < 0.001), indicating strengthened spatial dependence over time. The global spatiotemporal Moran’s I for 2013–2016 was 0.633 (Z = 31.933, *P* < 0.001), further confirming the aggregated pattern across space and time. Prostate cancer incidence showed a general increasing trend. Bayesian models revealed that spatial variation was dominated by unstructured spatial effects, with structured effects accounting for only 7.9% of the total spatial variance. This suggests that the observed variation was primarily driven by local, city-specific characteristics rather than broad regional clustering. Negative spatiotemporal interactions indicated that the evolution of incidence risk varied substantially across cities after adjusting for main spatial and temporal effects.

**Conclusions:**

Spatiotemporal interaction was identified as a major driver of incidence variation, which generally reduced relative risk in most areas but amplified it in a specific subset of cities. Therefore, policymakers should prioritize targeted interventions in these high-risk hotspots, rather than relying solely on generalized temporal trends for resource allocation.

**Supplementary Information:**

The online version contains supplementary material available at 10.1186/s12889-026-26619-7.

## Introduction

Prostate cancer ranks among the most prevalent male malignancies worldwide, imposing substantial burdens on public health systems and individual well-being [1]. Globally, incidence trends exhibit a consistent upward trajectory over the past two decades. In developed regions, rates remain persistently high. For instance, California (United States) reported 387,636 new cases between 2014 and 2021, including 27,938 distant metastases [2]; meanwhile, the United Kingdom witnessed age-standardized incidence rise from 109 to 159 per 100,000 during 2000 and 2021 [3]. However, a notable shift is occurring in regions historically characterized by lower incidence, particularly in Asia. This trend is exemplified by Singapore, which currently has the highest age-standardized rate in Asia [4], signifying a rapid epidemiological transition in the region.

**Fig. 1:**
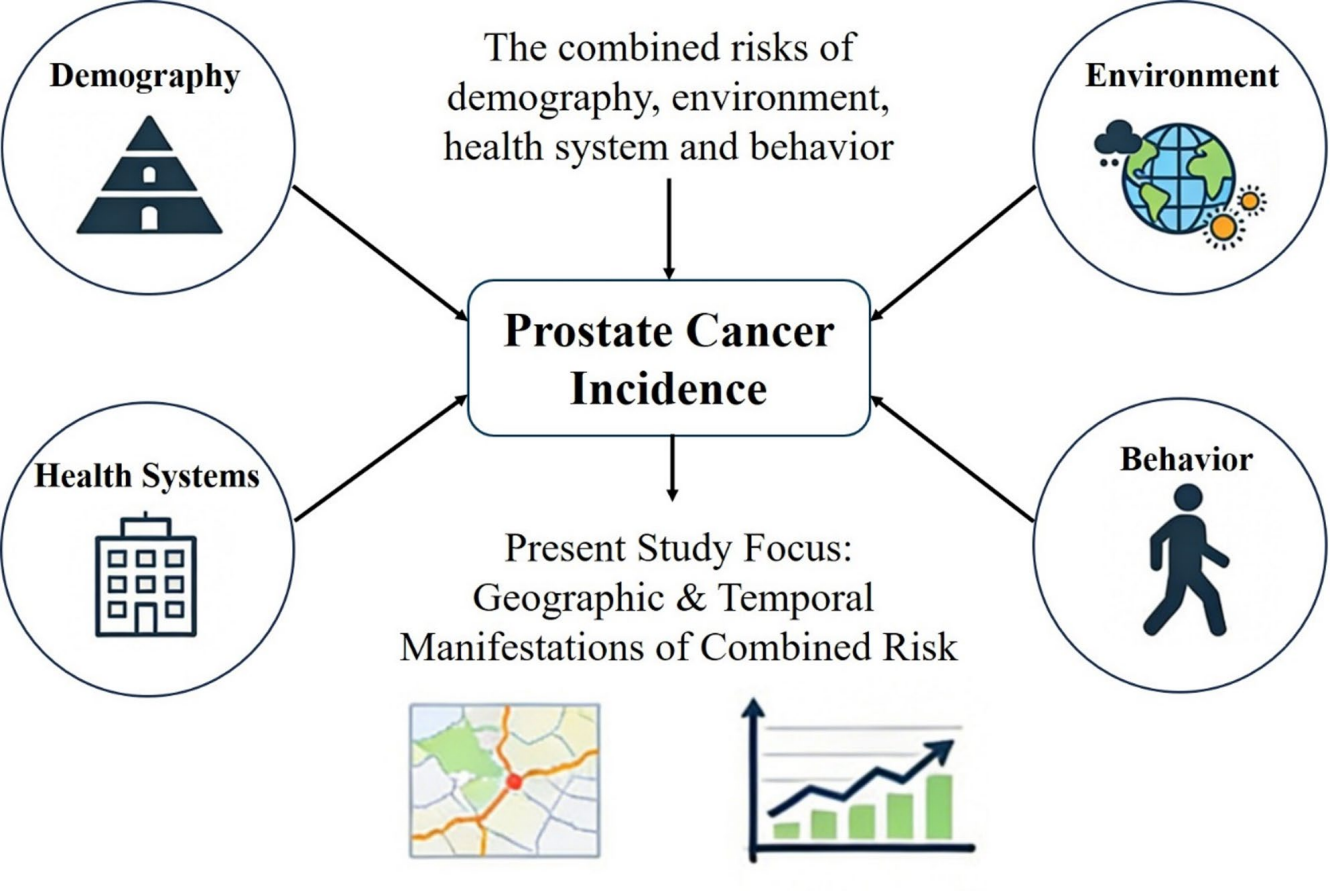
Conceptual framework of potential determinants of prostate cancer incidence. Note: This figure illustrates the theoretical factors influencing prostate cancer risk based on literature

In this global context, China’s evolving prostate cancer epidemiological profile carries significant implications for regional and global public health. Once characterized by incidence rates substantially lower than those in Western countries, China has witnessed a dramatic surge, making it a principal driver of male cancer morbidity [5]. This epidemiological transition is underpinned by a complex interplay of determinants, as illustrated in the conceptual framework in Fig. 1. These interacting domains include demographic shifts, such as rapid population aging [5, 6]; health system characteristics, notably the disparities in Prostate-Specific Antigen (PSA) screening and oncology care between urban and rural areas [7, 8]; behavioral changes, such as the adoption of Western dietary habits following socioeconomic development [9]. Consequently, a marked spatial pattern has emerged across the nation. An analysis of 182 counties (2014–2016) revealed a distinct geographic gradient: high-risk clusters were concentrated in the urbanized southeast, in contrast to with low-risk clusters in northern and central regions. This distribution aligns closely with regional variations in urbanization, economic development, and healthcare infrastructure [9]. Furthermore, while urban tertiary hospitals have improved early detection capabilities, delayed diagnoses in rural and underdeveloped regions continue to contribute to high national disability-adjusted life year (DALY) rates [10].

Despite growing recognition of prostate cancer’s rising burden in China, a critical knowledge gap persists regarding the spatiotemporal dynamics of the disease. Conceptually, spatiotemporal dynamics refer to the non-stationary dynamics where spatial disease patterns evolve differentially over time, rather than changing uniformly across all regions [11]. In the context of prostate cancer modeling, capturing this complexity is crucial because the primary drivers of incidence, such as populations aging, the uptake of PSA screening, and the adoption of Westernized lifestyles, do not diffuse evenly across China’s vast territory. Consequently, ignoring these spatiotemporal interactions risks oversimplifying disease dynamics and may lead to misguided resource allocation.

To address these challenges, selecting a robust methodological framework is essential. While classical frequentist methods, such as Maximum Likelihood Estimation (MLE), are widely used, they often struggle with the “small number problem” typical of cancer registry data in fine-grained areas. This limitation frequently results in unstable variance estimates and noise dominance [12]. In contrast, the Bayesian hierarchical framework offers distinct comparative advantages over MLE. By incorporating prior information and “borrowing strength” from neighboring areas and adjacent time points, Bayesian methods smooth unstable rates and provide more reliable estimates of the underlying risk [13, 14]. Moreover, Bayesian spatiotemporal interaction models (e.g., the Knorr-Held formulation) provide a solid theoretical basis for decomposing disease risk into structured spatial patterns (stable geography) [15], temporal trends, and the interaction term, specifically capturing the localized anomalies that deviate from the main trends [16, 17].

To bridge these critical gaps in fine-scale research, this study conducts a comprehensive analysis of prostate cancer incidence dynamics in China. Employing a Bayesian spatiotemporal interaction model at the city level, we systematically investigate geographic disparities with three specific objectives: (1) to map spatial distribution patterns and pinpoint high-risk clusters by employing both spatial and spatiotemporal autocorrelation analyses; (2) to characterize temporal trends and their spatial variations; and (3) to quantify the contribution of spatiotemporal interaction effects to the total observed variation. Through this approach, we aim to identify persist high-risk areas and decipher the underlying spatiotemporal structure, thereby generating actionable evidence to guide targeted prevention and the strategic healthcare resource allocation.

## Materials and methods

### Data sources

The study data were retrieved from the Annual Reports on Cancer Registration in China (2016–2019), published by the Chinese National Cancer Center [18–21]. These annual reports serve as the authoritative source for cancer surveillance data in China. Due to a standardized three-year reporting lag inherent to the registration system, the reports published between 2016 and 2019 correspond to the actual incidence data for the statistical years 2013–2016. Consequently, all subsequent references to “year” in this study denote these actual statistical years.

The analysis was restricted to the 2013–2016 period based on data quality and consistency criteria: (1) annual reports prior to 2013 contained prostate cancer incidence data from fewer than 200 reporting counties, and (2) these earlier years exhibited substantial data missingness that compromised analytical validity.

Geographically, this study covers cities across 31 provinces, autonomous regions, and municipalities in mainland China, including the Xinjiang Production and Construction Corps. Hong Kong, Macao, and Taiwan were excluded from the scope. Additionally, regions with no available data during the study period (e.g., Tibet) were excluded, as they were not covered by the Annual Reports. To maintain data integrity and avoid introducing bias arising from systematic differences between covered and uncovered regions, no imputation was performed for these missing areas. Ultimately, the final analytical dataset comprised 132 cities, encompassing a total of 248 counties and districts.

### Data processing and outcome variable

The annual reports provided crude incidence rates, as well as age-standardized rates (using both the Chinese standard and the world standard) at the county level. However, preliminary modeling using these granular county-level data revealed significant instability, primarily attributable to the “small numbers problem” common in fine-scale epidemiological studies. Furthermore, inconsistent reporting practices across counties resulted in substantial temporal gaps.

To address these limitations and ensure robust parameter estimation, we aggregated the data to the city level. For each city and each year (2013–2016), we extracted the reported number of new prostate cancer cases ($\:{O}_{it}$) and the corresponding total male population ($\:{N}_{it}$) from all constituent counties within that city’s administrative boundaries. This aggregation strategy ensures that city-level estimates are derived directly from the complete reporting of all constituent counties, thereby significantly enhancing data reliability and statistical stability.

While city-level crude rates provide a direct measure of disease burden, they are inherently confounded by the varying age structures across different cities, particularly for prostate cancer, which is strongly age-dependent. To rigorously address this confounding and facilitate valid spatial-temporal comparisons, this study utilizes the Standardized Incidence Ratio (SIR) as the primary outcome variable, calculated via indirect standardization. Due to the unavailability of detailed age-specific population data at the individual city level, we employed a demographic estimation strategy. We applied the annual age composition (proportion of 0–14, 15–64, and ≥ 65 years) of the corresponding province, obtained from China Statistical Yearbooks, to each city’s total population to estimate the city-level age-specific denominators.

The expected number of cases ($\:{E}_{it}$) for each city was then calculated by applying the national age-specific incidence rates [22] to these estimated city populations. Finally, the SIR was defined as the ratio of the observed number of cases to the expected number of cases: $\:SIR=\frac{{O}_{it}}{\:{E}_{it}\:}$. An SIR > 1 indicates a higher-than-expected risk relative to the national average, after adjusting for age structure.

### Statistical analysis

#### Spatial exploratory analysis

Spatial autocorrelation analysis was employed to uncover the spatial distribution characteristics of prostate cancer incidence in China and the degree of correlation between the incidence risk of various regions and their surrounding areas was quantified. The analysis encompasses both global spatial autocorrelation analysis and the analysis of local hotspot areas (Getis-Ord Gi*). All space-related research and mapping were completed in ArcGIS10.8.

##### Global Moran’s I

The global Moran’s I index [-1, 1] was used to detect the overall spatial distribution pattern of prostate cancer incidence risk across cities in China [23]. A positive Moran’s I index signifies spatial positive autocorrelation (similar incidence risks in adjacent cities), while a negative index suggests spatial negative autocorrelation (dissimilar incidence risks in adjacent cities). The formula is:

1 $$\:\begin{array}{c}Mora{n}^{{\prime\:}}s\:I=\frac{n\sum\:_{i=1}^{n}\sum\:_{j=1}^{n}{W}_{ij}\left({z}_{i}-\stackrel{-}{z}\right)\left({z}_{j}-\stackrel{-}{z}\right)}{\sum\:_{i=1}^{n}\sum\:_{j=1}^{n}{W}_{ij}\sum\:_{i=1}^{n}{\left({z}_{i}-\stackrel{-}{z}\right)}^{2}}\end{array}$$

Here, *n* denotes the total number of cities; $\:{z}_{i}$ and $\:{z}_{j}$ represent the incidence rates of prostate cancer for cities *i* and *j*, $\:\stackrel{-}{z}$ indicates the average incidence rate of prostate cancer across the nation. $\:{W}_{ij}$ corresponds to the spatial weight matrix reflecting the spatial relationships between various cities in China, which is defined based on adjacency/contiguity between the cities.

##### Local Moran’s I

The analysis was further performed using local indicators of spatial association (LISA) to specifically detect local spatial instability and outliers [24]. The LISA quantifies the spatial distribution between a specific city and its neighboring cities, enabling the identification of four types of local spatial patterns: High-High (HH) and Low-Low (LL) clusters, and distinctively, spatial outliers: High-Low (HL, high-incidence cities surrounded by low-incidence neighbors), and Low-High outliers (LH, low-incidence cities surrounded by high-incidence neighbors). This method is essential for identifying “transition zones” or local anomalies that global clustering methods might overlook.

##### Getis-Ord Gi* Statistics 

To complement the LISA analysis, the Getis-Ord Gi* statistic was employed to visualize the intensity and broader regional extent of high-value concentrations (hotspots) and low-value concentrations (coldspots). Unlike LISA, which emphasizes specific outlier relationships, Gi focuses on the cumulative magnitude of values in a local neighborhood relative to the global average [25, 26]. The formula is:

2 $$\:\begin{array}{c}{G}_{i}^{*}=\frac{\sum\:_{j=1}^{n}{w}_{ij}{x}_{j}-\frac{\sum\:_{j=1}^{n}{x}_{j}}{n}\sum\:_{j=1}^{n}{w}_{ij}}{\sqrt{\frac{\sum\:_{j=1}^{n}{x}_{j}^{2}}{n}-({\frac{\sum\:_{j=1}^{n}{x}_{j}}{n})}^{2}}\sqrt{\frac{n\sum\:_{j=1}^{n}{w}_{ij}^{2}-{\left(\sum\:_{j=1}^{n}{w}_{ij}\right)}^{2}}{n-1}}}\end{array}$$

In Eq. (2), the parameters are defined as follows: $\:{x}_{j}$ represents the incidence rate of prostate cancer in city *j*, $\:{w}_{ij}$ denotes the spatial weight matrix between cities *i* and *j* (used to quantify the strength of spatial association between the two cities).

While related, the LISA and Getis-Ord Gi* Statistics address distinct spatial questions. Gi* statistics quantify regional risk aggregation (global spatial autocorrelation), whereas Local Moran’s I (LISA) delineate site-specific deviations (local heterogeneity). A municipality simultaneously classified as a Gi* hotspot and a High-Low outlier thus represent focal risk intensification nested within a broader high-risk cluster—a spatial configuration indicating local deviation embedded within global aggregation. These divergent classifications reveal nested spatial heterogeneity rather than contradictory findings, reflecting the methodological complementarity of global and local indicators.

##### Spatiotemporal autocorrelation analysis

To quantify the spatiotemporal autocorrelation of prostate cancer incidence, the spatiotemporal Global Moran’s I (MoranST) [11] was calculated. The equation was as follows:

3 $$\:\begin{array}{c}MoranST=\frac{nT\sum\:_{i=1}^{n}\sum\:_{t=1}^{T}\sum\:_{j=1}^{n}\sum\:_{s=1}^{T}{\stackrel{\sim}{w}}_{(it,js)}({y}_{it}-\stackrel{-}{y})({y}_{js}-\stackrel{-}{y})}{\sum\:_{i=1}^{n}\sum\:_{t=1}^{T}\sum\:_{j=1}^{n}\sum\:_{s=1}^{T}{\stackrel{\sim}{w}}_{(it,js)}\sum\:_{i=1}^{n}\sum\:_{t}^{T}{({y}_{it}-y)}^{2}}\end{array}$$

Here, $\:\stackrel{-}{y}$ denotes the mean of the observed values $\:{y}_{it}$ (prostate cancer incidence) across all cities i and years t, n is the number of cities, T is the number of years, and $\:\stackrel{\sim}{w}$ *(i*,* t; j*,* s)* is the spatiotemporal weight matrix (incorporating both geographic adjacency between cities and temporal continuity across years).

#### Spatial weights matrix

To quantify spatial dependence, a first-order Queen contiguity matrix was constructed. In this binary symmetric matrix $\:W=\left\{{w}_{ij}\right\}$, an element $\:{w}_{ij}$ is assigned a value of 1 if city *i* and city *j* share any common boundary point (including edges or vertices), indicating they are neighbors; otherwise, $\:{w}_{ij}=0$. The matrix was then row-standardized so that each row summed to 1. We selected the Queen contiguity matrix over other specifications because because our study focuses on contiguous administrative units (cities in mainland China), where contiguity-based adjacency more intuitively reflects actual geographical proximity and potential shared risk factors (e.g., environmental exposures, socioeconomic connections) between neighboring administrative regions.

#### Bayesian spatiotemporal model

To explore the spatiotemporal dynamics of prostate cancer incidence risk across various cities in China, this study employs a Bayesian spatiotemporal modeling framework. The model integrates spatial effects, temporal effects, and their interaction to quantitatively analyze the dynamic characteristics of prostate cancer incidence risk.

##### Likelihood and distributional assumption

The observed number of prostate cancer cases in city *i* during year *t*, denoted as $\:{Y}_{it}$, is modeled using a Poisson distribution. As correctly noted in standard disease-mapping formulations, the likelihood is defined as:

4 $$\:\begin{array}{c}{Y}_{it}\:\mid\:\:{E}_{it}{\theta\:}_{it}\sim{Poisson}\left({E}_{it}{\theta\:}_{it}\right)\end{array}$$

Here, $\:{Y}_{it}$ is the observed count of new prostate cancer cases. $\:{\theta\:}_{it}$ is the relative risk (RR) to be estimated, representing the smoothed SIR. $\:\:{E}_{it}$ is the expected number of cases, acting as the offset in the model.

Calculation of the Expected Number of Cases ($\:{E}_{it}$).

Consistent with the indirect standardization method, $\:{E}_{it}$ was calculated to adjust for the varying age structures across cities. Specifically, it represents the summation of the product of the city’s estimated age-specific population ($\:{N}_{it,\:k}$) and the national age-specific incidence rates ($\:{R}_{std,\:k}$):

5 $$\:\begin{array}{c}{E}_{it}=\sum\:_{k}({N}_{it,\:k}\:\times\:\:{R}_{std,\:k})\end{array}$$

Here, $\:{N}_{it,\:k}$ was estimated based on the age composition (0–14, 15–64, 65+) of the province to which city *i* belongs, and $\:{R}_{std,\:k}$ corresponds to the national reference rates from 2015 [22]. This formulation ensures that the estimated relative risk $\:{\theta\:}_{it}$ reflects the risk level relative to the national average, after controlling for local demographic differences.

##### Design of the model structure

To systematically identify the spatiotemporal characteristics of prostate cancer incidence, this study sequentially constructed and compared three types of models: the first type is a pure Bayesian spatial model without considering the temporal effects of prostate cancer incidence (see Eq. 6):

6 $$\:\begin{array}{c}\mathrm{log}\left({\theta\:}_{i}\right)=\alpha\:+{u}_{i}+{v}_{i}\end{array}$$

The second type is a pure Bayesian temporal model without considering the spatial effects of prostate cancer incidence (see Eq. 7):

7 $$\:\begin{array}{c}\mathrm{log}\left({\theta\:}_{i}\right)=\alpha\:+{\gamma\:}_{t}+{\varphi\:}_{t}\end{array}$$

The third type is Bayesian spatiotemporal interaction model that accounts for the variation of spatial effects over time in prostate cancer incidence (see Eq. 8).

8 $$\:\begin{array}{c}\mathrm{log}\left({\theta\:}_{i}\right)=\alpha\:+{u}_{i}+{v}_{i}+{\gamma\:}_{t}+{\varphi\:}_{t}+{\delta\:}_{it}\end{array}$$

In these models, the parameter α represents the intercept, reflecting the natural logarithm of the average relative risk of prostate cancer over the study period. The $\:({u}_{i}+{v}_{i})$ represents both structured and unstructured spatial effects, while the parameter ($\:{\gamma}_{t}+{\varphi}_{t}$) represents both structured and unstructured temporal effects.

The structured spatial effect captures residual spatially smooth patterns of prostate cancer risk that vary gradually across neighboring cities after accounting for the covariates included in the model. It reflects unobserved heterogeneity associated with underlying geographical processes (which may potentially include unmeasured factors such as regional environmental exposures or healthcare accessibility patterns) that create spatial clustering, wherein adjacent areas tend to have similar risk levels. Unstructured spatial effect represents random variation between cities that cannot be explained by geographical proximity. It accounts for unmeasured city-specific factors (e.g., local idiosyncrasies or noise) that cause risk to deviate independently from neighboring areas. Structured temporal effect captures smooth temporal trends in prostate cancer risk over the study period, potentially reflecting gradual changes in unobserved determinants, such as evolving diagnostic practices or shifting population-level risk factors. Unstructured temporal effect represents year-specific random fluctuations that deviate from the smooth temporal trend, accounting for irregular annual variations. Additionally, the parameter $\:{\delta\:}_{it}$ indicates the spatiotemporal interaction effect, quantifying the deviation in risk for a specific city during a specific year from what would be expected based on the main spatial and temporal effects alone. A positive value indicates that the observed risk exceeds the expectation derived from combining the city’s average spatial risk and the year’s temporal trend; a negative value indicates lower-than-expected risk.

The spatiotemporal interaction term $\:{\delta\:}_{it}$ characterizes “abnormal fluctuations in risk of a specific region at a specific time”, representing the joint effect of regional characteristics and temporal trends. Following the classification by Knorr-Held [15], this term was specified as a Type IV interaction (Structured Space × Structured Time), modeled as the product of a structured spatial effect (Besag) and a structured temporal effect (AR(1)). This interaction term follows a normal distribution $\:{\delta\:}_{it}$~N (0, $\:{{{\upsigma\:}}_{\delta\:}}^{2}$). A penalized complexity (PC) prior was assigned to its precision parameter $\:{\tau\:}_{\delta\:}$ to flexibly control the variation range of the interaction effect.

#### Prior selection for parameters and hyperparameters

##### Fixed effects

For the intercept term α, a non-informative prior N (0, $\:{10}^{6}$) was adopted. The large variance of this prior provides sufficient degrees of freedom for the parameter, minimizing the interference of subjective information on estimation and ensuring that results are more data-driven.

##### Spatial effects

The BYM model decomposes spatial effects into “structured-unstructured dual components” to distinguish the contributions of “spatial dependence between regions” and “independent variation within individual regions” to disease risk [27]. Structured component $\:{u}_{i}$ characterizes the spatial distribution between adjacent cities (e.g., disease risks in geographically neighboring regions tend to be more similar). It follows an intrinsic conditional autoregressive (ICAR) distribution, whose core assumption is that “the effect value of a city depends on the mean effect value of its surrounding cities”, which is consistent with the theoretical expectation of “spatial aggregation” in epidemiology. Unstructured component $\:{v}_{i}$ captures the independent random variation (or unstructured variability) of individual regions (e.g., localized factors not shared with neighboring areas). It follows a normal distribution $\:{v}_{i}$~N (0, σ_v_²), where σ_v_² denotes the variance of the unstructured component. The precision parameters of these two components—τ_u_=1/σ_u_² and τ_v_=1/σ_v_²—were both assigned a weakly informative prior loggamma (1, 0.0005). This prior is widely used in spatial modeling, as it balances the uncertainty of parameter estimation by preventing divergent parameter estimates while preserving the ability to truly characterize spatial variability from data. However, the BYM model suffers from an identifiability issue with the precision parameters τ_u_ and τ_v_: the posterior estimates of these two parameters tend to interfere with each other, potentially leading to ambiguous interpretations of spatial effects. To address this limitation, the BYM2 model was further employed in this study for optimization [28]. Through reparameterization, the spatial effect was expressed as:

9 $$\:\begin{array}{c}{b}_{i}=\frac{1}{\sqrt{{\tau\:}_{b}}}(\sqrt{1-w}{v}_{i}+\sqrt{w}{u}_{i}^{*})\end{array}$$

where marginal precision $\:{\tau}_{b}$ is a parameter controlling the overall variability of spatial effects, it directly determines the global fluctuation amplitude of spatial effects. A *PC prior* (*U* = 1, *α* = 0.01) [293] was assigned to the precision $\:{\tau}_{b}$. This PC prior [29] implies that “the probability of the marginal variance of spatial effects being greater than 1 is only 1%”. It avoids overestimating spatial variability through strong penalization, ensuring that the model’s characterization of spatial risk distribution aligns with actual data patterns.

The mixing parameter *w* quantifies the contribution ratio of the standardized structured component to the spatial effect; a value indicating a smaller structured component suggests a more prominent role of independent local variability (or unstructured noise). A *pc(U = 0.5*,* α = 0.5)* prior was set for *w*, which means “the probability that the contribution ratio of the structured component exceeds 50% is 50%”. This weak penalization balances the interpretive weights of the two types of spatial effects and prevents prior information from overly dominating parameter estimation.

Standardized structured component $\:{u}_{i}^{*}$ derived by standardizing the structured component of the original ICAR distribution, it eliminates scale dependence and makes the interpretation of the “contribution ratio” of the mixing parameter w more intuitive. No additional prior was required for $\:{u}_{i}^{*}$.

##### Temporal effects

To capture the smoothness or dynamic correlation of disease risk over time, temporal effects were modeled using random walk (RW1, RW2) and autoregressive (AR(1), AR(2)) process. For the precision parameters $\:{\tau}_\upgamma$ (associated with the temporal structured effect $\:{\gamma}_{t}$) and $\:{\tau}_{\phi\:}$ (associated with the independent temporal effect $\:{\varphi}_{t}$), either a loggamma (1, 0.0005) prior or a PC prior was adopted. For the autocorrelation coefficient ρ of the AR process, either a normal distribution N (0, 0.15) or PC prior (pc.cor0) was used. These priors control the strength of temporal dependence to avoid overfitting.

#### Bayesian model calculation and evaluation

The posterior distribution of parameters is estimated using the INLA (integrated nested Laplace approximations) method. This method quickly solves the posterior distribution through Laplace approximation, and its computational efficiency exceeds that of the traditional MCMC method to a significant extent, with similar precision. The model implementation is based on the R-INLA package (http://www.r-inla.org/), and the running environment is RStudio 4.5.1. To select the best-fitting model, the deviance information criterion (DIC) and the Watanabe-Akaike information criterion (WAIC) were employed to compare the merits and demerits of the models [30–32]. The use of two criteria provides a robust assessment, leveraging the widespread adoption of DIC in spatial epidemiology and the theoretically rigorous predictive accuracy estimation of WAIC. The model achieving the lowest values on both DIC and WAIC was selected as optimal. Additionally, posterior predictive checks using Probability Integral Transform (PIT) values were performed to verify model calibration.

#### Sensitivity analysis

To assess the robustness of the model results to the specification of PC priors, a systematic sensitivity analysis was conducted by varying the hyperparameters *U* and *α* across all PC priors in the model. Specifically, for the precision parameters ($\:{{\uptau}}_{b}$, ${{\uptau}}_{{\upgamma\:}}$, $\:{\tau}_{\phi\:}$, $\:{{\uptau}}_{{\updelta\:}}$), we explored the range of variance threshold *U* from 0.5 to 2 and the probability of exceeding the threshold α from 0.01 to 0.1, covering both constrained and relax prior setting. For the BYM2 mixing parameter ω, we adjusted the contribution ratio threshold *U* within the range of 0.3 to 0.7 to examine the sensitivity of results to the weighting of structured versus unstructured spatial effects. For the AR(1) autocorrelation coefficient ρ1, we modified the correlation strength threshold *U* from 0.1 to 0.4 with a fixed α of 0.2, to verify the impact of prior assumptions on temporal dependence patterns. All other model parameters were fixed at their baseline specifications during the sensitivity analysis to isolate the impact of each prior adjustment. We evaluated model robustness by comparing model fit indices (DIC, WAIC).

## Results

### Spatial and temporal characteristics

**Fig. 2:**
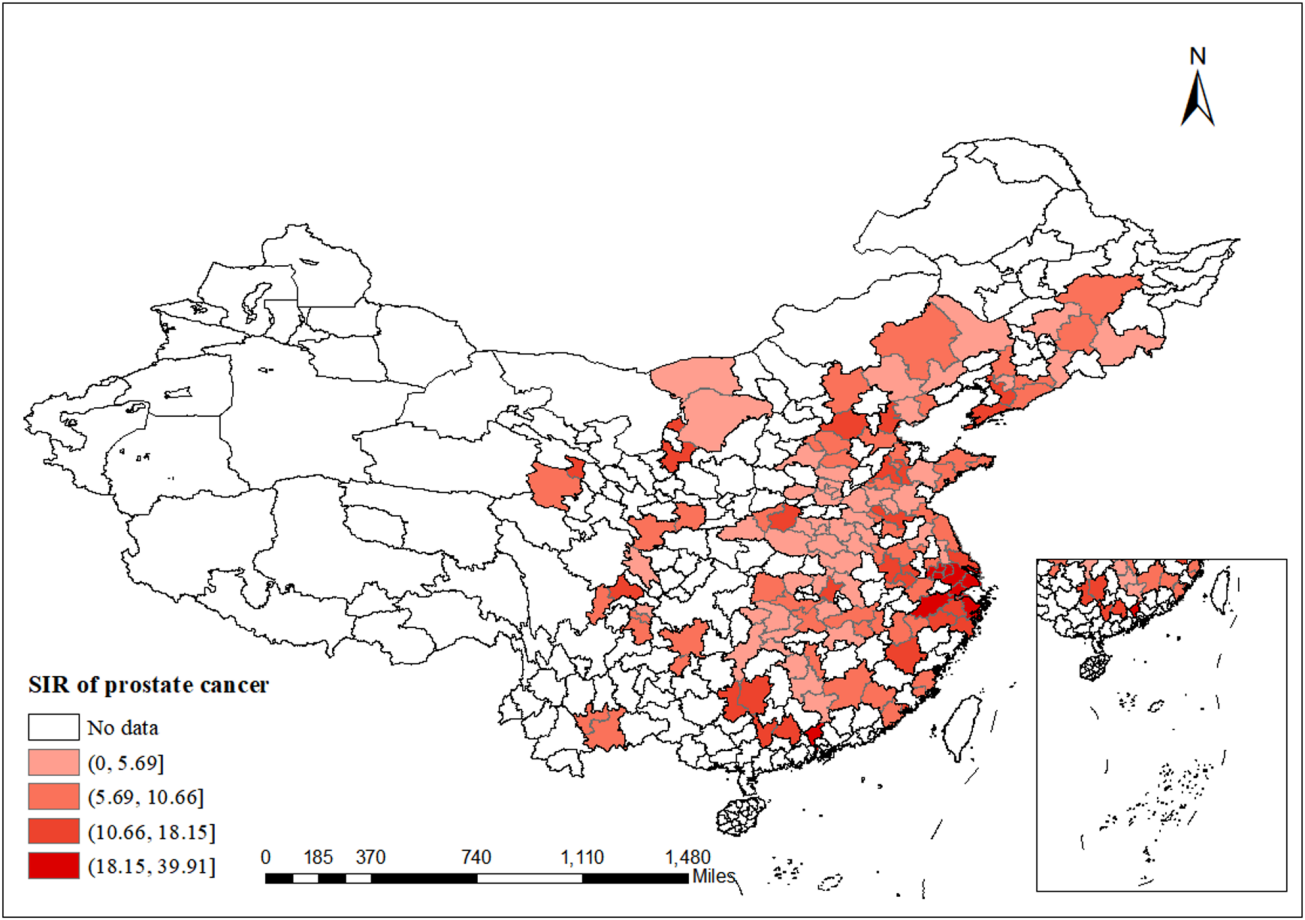
Distribution of SIR (/100,000 population) of prostate cancer in 2016. Note: White areas indicate regions with no data available in the Annual Reports on Cancer Registration

Spatial data from 2013 to 2016 (Fig. 2, Supplementary Figs. 1 A-C) showed significant and persistent geographical variation in prostate cancer incidence across the study area. High-incidence cities were consistently concentrated in the southeastern coastal regions, while moderate-incidence areas spanned major eastern and central provinces, maintaining a stable distribution throughout the study period. Conversely, low-incidence areas persisted in the northwestern and northern provinces, showing no major spatial shifts over the four years. Overall, the spatial distribution followed a distinct macro-pattern characterized by higher levels in the eastern and southern regions, contrasting with lower levels in the western and northern areas.

**Fig. 3:**
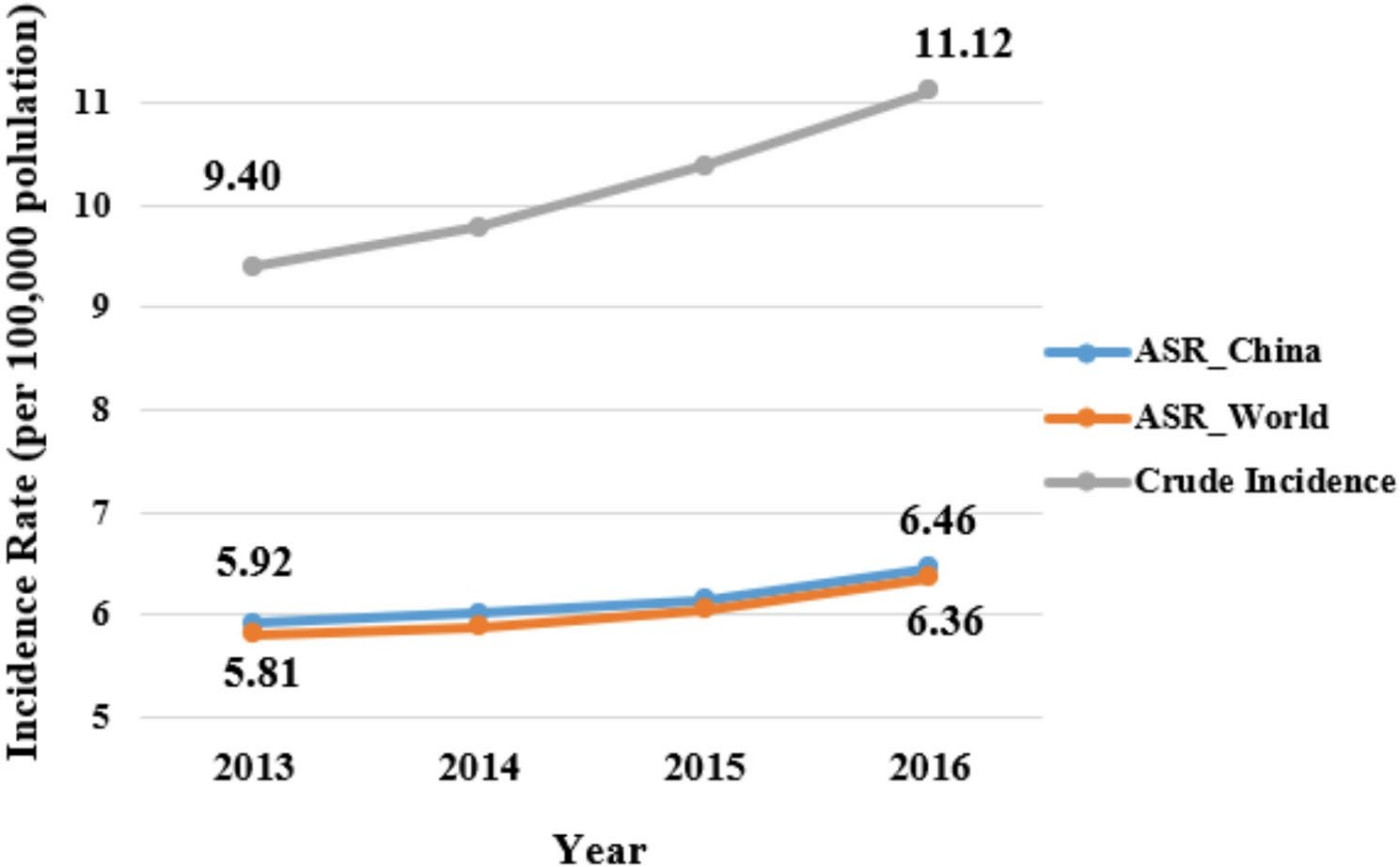
The trend of crude incidence, ASR_China, and ASR_World of prostate cancer from 2013 to 2016 (/100,000 population)

Temporally, the incidence rate exhibited a continuous upward trend between 2013 and 2016 (Fig. 3). Most notably, the crude incidence rate increased with the most prominent growth rate, averaging an annual increase of approximately 5.78%. Concurrently, the age-standardized rates (ASR_China and ASR_World) showed similar but more moderate trajectories, maintaining annual growth rates between 2.95% and 3.05% [18–21].

### Results of spatial and spatiotemporal autocorrelation analysis

Global spatial autocorrelation analysis (Supplementary Table S1) confirmed a significant positive spatial dependency for prostate cancer incidence. The Global Moran’s I values were consistently positive and increased annually from 0.141 in 2013 to 0.173 in 2016 (all *P* < 0.0001). This trend indicates not only that high-incidence areas are geographically clustered but also that the strength of this spatial association has intensified over time. Furthermore, the spatiotemporal autocorrelation analysis yielded a Global Moran’s I of 0.633 for the entire 2013–2016 period (*P* < 0.001, Z = 31.933). This value is significantly higher than the purely spatial Moran’s I observed in individual years, suggesting a profound spatiotemporal inertia. This finding implies that the agglomeration of prostate cancer incidence is driven by both geographical proximity and temporal continuity: high-incidence areas tend to cluster spatially and persist in a high-risk state over consecutive years, thereby forming stable spatiotemporal hotspots.

**Fig. 4:**
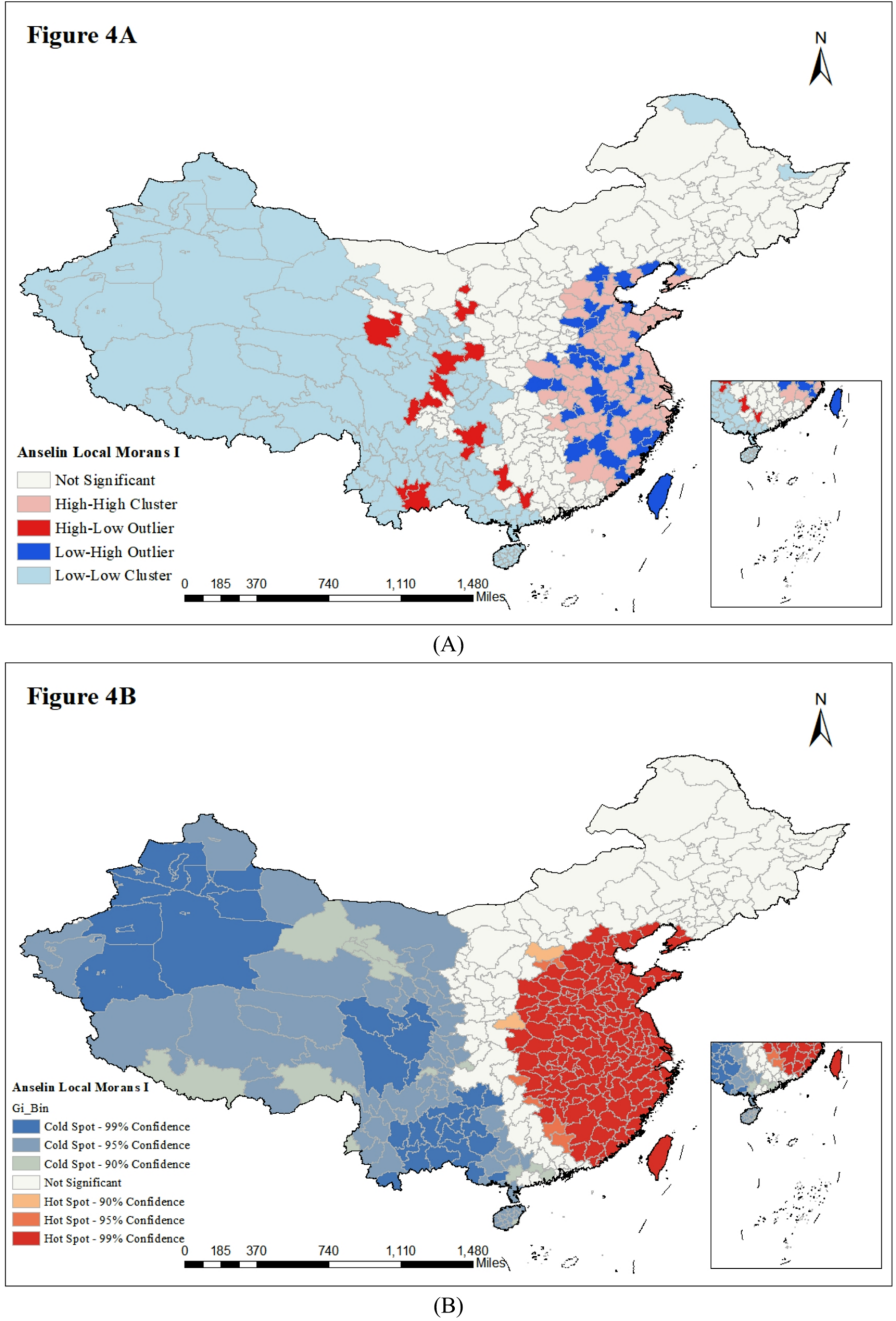
The map of (**A**) cluster and outlier analysis and (**B**) hotspot analysis of SIR (/100,000 population) of prostate cancer in China in 2016

Local clustering analysis (Fig. 4A, Supplementary Figs. 2 A-C) detailed aggregation: from 2013 to 2016, southeastern coastal regions had stable “High-High” clustering (high incidence + distinct spatial clustering). “High-Low” outliers were rare; some central/western regions had “Low-High” outliers in specific years (low incidence but influenced by nearby high-incidence areas).

Consistent with these findings, the Hotspot analysis (Fig. 4B, Supplementary Figs. 3 A-C) identified the southeastern coast as a persistent hotspot for prostate cancer. Crucially, the statistical confidence of these hotspots increased over the study period, reflecting a strengthening of high-incidence significance. In contrast, coldspots were predominantly centered in the western and northern provinces, characterizing regions of clustered low risk.

### Results of Bayesian spatiotemporal modeling

#### Model selection

A series of candidate models were systematically evaluated, ranging from pure spatial and temporal formulations to complex spatiotemporal interaction models. Based on the goodness-of-fit criteria (DIC and WAIC) and parameter stability, the spatiotemporal interaction model incorporating the BYM2 spatial structure and Penalized Complexity (PC) priors (Model 12) was selected as the optimal model. This model demonstrated the best balance between model fit and parsimony, and sensitivity analyses confirmed its robustness to hyperparameter variations. Detailed comparisons of candidate models, step-by-step selection procedures, and sensitivity analysis results are fully documented in the Supplementary Materials (Supplementary Text 1 and Supplementary Tables S2–S4).

#### Results of the Bayesian spatiotemporal interaction model

Based on the BYM2 model parameterization, the posterior mean of the mixing parameter (ω) was 0.089. This indicates that only 8.9% of the total spatial variance is attributable to spatially structured correlation, while the vast majority is driven by unstructured variation (random noise), suggesting highly localized heterogeneity in the disease burden.

The findings of the optimal Model 12 are presented in Table 1. Specifically, the posterior mean of the precision parameter shared by the spatial structural effect and the non-spatial effect is 5.777 (95%CI: 4.306, 7.659); the posterior mean of the spatial mixing parameter ω is 0.079 (95%CI: 0.005, 0.317), corresponding to a contribution rate of spatial structural effect to the total spatial variation of 7.9%. This suggests that the variation attributed to the spatial structural effect accounts for 7.9% of the total spatial variation, indicating that the spatial variation in the risk of prostate cancer incidence is primarily driven by unstructured spatial variation.

**Table 1:** The results of the bayesian space-time interaction model

Models	Spatio-temporal independence effect	Hyperparameters of space-time effect	Prior distribution	Parameter posterior estimation			DIC	WAIC
				Posterior mean	95%CI	SD		
Model 11	BYM+RW1 + iid+Besag (spatiotemporal interaction)	$\:{\tau}_{u}$	Default prior, the spatial effect prior is the same as Model 1, and the temporal effect prior is the same as Model 3	2204.950	(147.540, 8623.200)	2420.000	4299.32	4343.61
		$\:{\tau}_{v}$		8.980	(2.910, 20.380)	457.000		
		$\:{\tau}_{y}$		20529.84	(1162.120, 81893.940)	23,100		
		$\:{\tau}_{\phi\:}$		980.170	(174.680, 3006.270)	769.000		
		$\:{\tau}_{\delta\:}$	loggamma (1, 0.0005)	1.900	(1.570, 2.260)	0.175		
Model 12 (Type IV)	BYM2 + AR(1) + iid+ Besag (spatiotemporal interaction)	$\:{\tau}_{b}$	Prior of PC, the prior of spatial effects is the same as that of Model 2, and the prior of temporal effects is the same as that of Model 8	5.777	(4.306, 7.659)	0.853	4277.04	4298.94
		$\omega$		0.079	(0.005, 0.317)	0.084		
		$\:{\tau}_{y}$		122.585	(34.145, 322.086)	76.502		
		$\rho{1}$		0.0001	(-0.009, 0.091)	0.046		
		$\:{\tau}_{\phi\:}$		86.804	(41.858, 165.411)	31.858		
		$\:{\tau}_{\delta\:}$	pc.prec (1, 0.01)	2.012	(1.709, 2.355)	0.164		

*BYM* Besag-York-Mollié model (a spatial effect model combining structured and unstructured spatial variation), *BYM2* Besag-York-Mollié 2 model (an updated version of BYM, optimizing the mixing of structured/unstructured spatial variation), *AR(1)* First-order autoregressive model (used to characterize temporal autocorrelation), *AR(2)* Second-order autoregressive model (a temporal dependence model where the current value of a time series depends linearly on its values at the previous 2 periods, *iid* Independent and identically distributed (referring to unstructured random effects)

$\:{\tau}_{u}$: Precision parameter of the unstructured spatial effect

$\:{\tau}_{v}$: Precision parameter of the structured spatial effect

$\:{\tau}_{y}$: Precision parameter of the temporal structured effect

$\rho{1}$, $\rho{2}$: Autocorrelation coefficients (for temporal/space-time dependence)

$\:{\tau}_{\phi}$: Precision parameter of the temporal unstructured (iid) effect

$\:{\tau}_{\delta\:}$: Precision parameter of the spatiotemporal interaction effect

$\:{\tau}_{b}$: Marginal accuracy of the BYM2 model

$\omega$: Mixing parameter of the BYM2 model (proportion of structured spatial variation)

*PC* Penalized Complexity, *SD* Standard Deviation, *DIC* deviance information criterion, *WAIC* Watanabe-Akaike information criterion

For the temporal structural effect of prostate cancer, the posterior mean of the precision parameter is 122.585 (95%CI: 34.145, 322.086), and the posterior mean of the first-order temporal autocorrelation coefficient is 0.0001 (95%CI: -0.009, 0.091), showing no significant correlation in the risk of prostate cancer incidence over time. Additionally, the posterior mean of the precision coefficient for the temporal unstructured spatial effect is 86.804 (95%CI: 41.858, 165.411), further indicating that the temporal variation in prostate cancer primarily originates from both temporal structural and unstructured spatial effects, with no significant association between the incidence risks at adjacent time points.

**Fig. 5:**
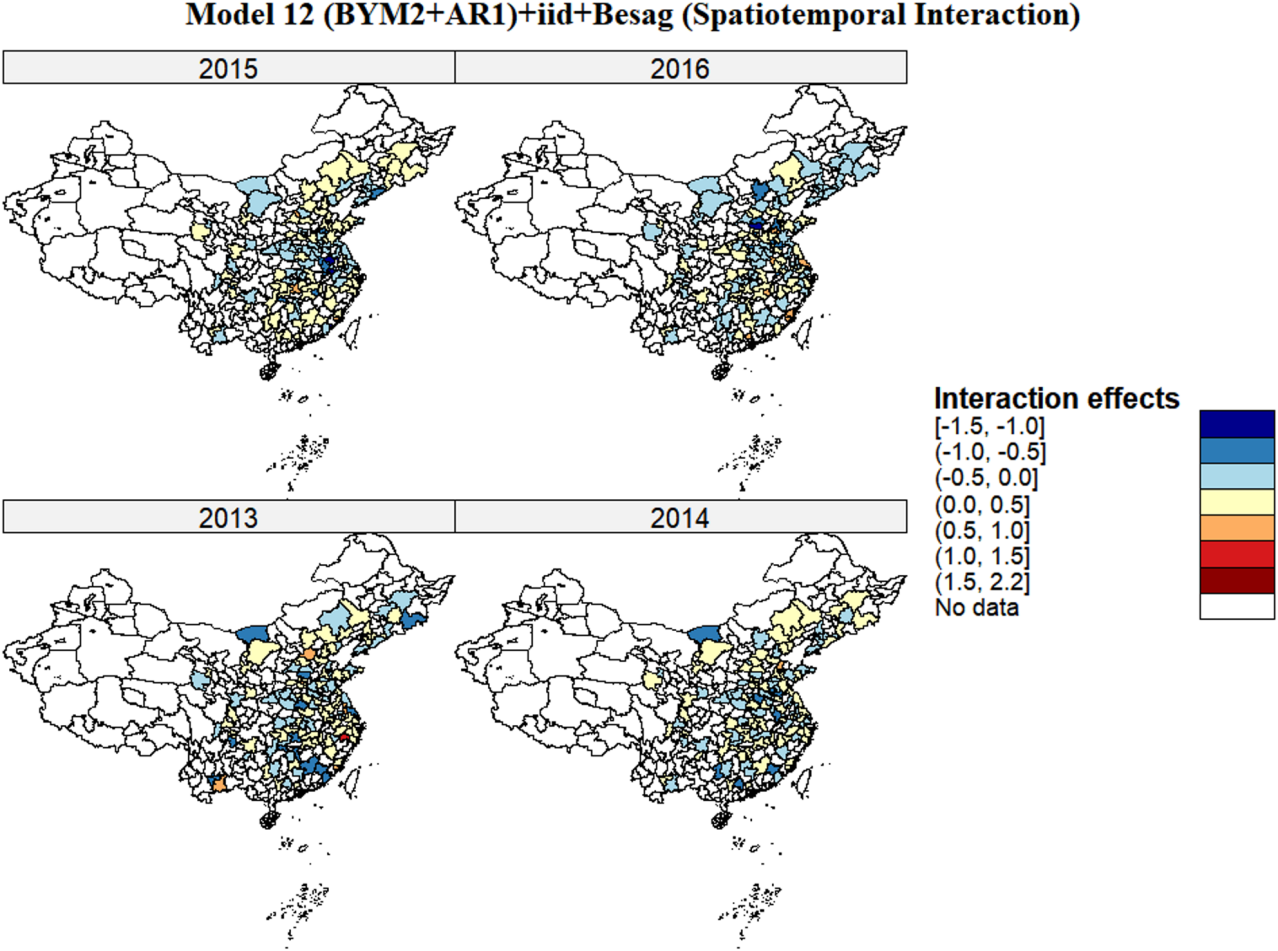
Posterior mean of spatiotemporal interaction effects ($\:{\delta\:}_{it}$) of prostate cancer in China in model 12. Note: Red indicates higher interaction effects, and Blue indicates lower interaction effects

The posterior mean of the precision parameter for the spatiotemporal interaction effect for prostate cancer is 2.012 (95%CI: 1.709, 2.355). As precision is the reciprocal of variance, this value indicates that after controlling for the main spatial and temporal effects, the spatiotemporal interaction effect of prostate cancer incidence exhibits substantial variability across cities. Specifically, a relatively low precision value implies high variability in the magnitude and direction of the spatiotemporal interaction effect among different cities in China during 2013–2016. The posterior distribution of the spatiotemporal interaction effect values at the city level is presented in Fig. 5. During the period 2013–2016, the majority of regions in China exhibited a negative spatiotemporal interaction effect, with the dark blue areas (effect value range: [-1.5, -1.0]) showing the most pronounced negative effects, indicating that the risk of prostate cancer incidence in these areas was lower than the additive expectation of independent spatial and temporal effects. Light blue areas (effect ranges: [-1.0, -0.5] and [-0.5, 0.0]) displayed relatively weaker negative interactions, although their incidence risk still fell below the additive expected level. Concurrently, a small subset of regions showed positive spatiotemporal interaction effects: Yellow (effect value range: (0.0, 0.5]), orange (effect value range: (0.5, 1.0]) and dark red areas (effect range: (1.0, 1.5] and (1.5, 2.2]) had incidence risks exceeding the additive sum of spatial and temporal effects alone. PIT histogram further confirmed the model’s adequacy, despite some expected deviations due to data sparsity in low-incidence regions (Supplementary Fig. 4).

## Discussion

This study provides a coherent portrait of the dynamic landscape of prostate cancer in China from 2013 to 2016. The identified spatiotemporal patterns reveal a distinct southeast-to-northwest gradient that closely parallels China’s well-documented regional disparities in socioeconomic development and healthcare resource distribution [9]. While this descriptive model did not explicitly quantify the contribution of specific covariates, the persistence of these high-risk clusters in economically developed coastal regions is consistent with the literature linking prostate cancer incidence to lifestyles, aging populations, and higher accessibility to PSA screening [33–35]. Furthermore, the temporal stability of this spatial hierarchy suggests that the disease burden is likely to these structural sociodemographic factors rather than transient environmental fluctuations. Consequently, the observed patterns serve as a hypothesis-generating foundation, highlighting areas where these potential drivers may be most active.

To unravel the specific texture of this spatial gradient, the dual-statistic approach provided complementary insights. While Getis-Ord Gi* analysis successfully delineated broad regional hotspots that characterize the macroscopic disease burden, the LISA analysis specifically pinpointed spatial outliers to reveal microscopic anomalies. Crucially, the overlap where specific cities are identified as outliers by LISA despite being located within broader Gi hotspots does not imply contradiction. Rather, it highlights internal heterogeneity, indicating that localized peaks of intense risk often drive the broader regional averages. The observed “high-Southeast, low-Northwest” spatial gradient aligns closely with regional disparities in socioeconomic development and lifestyle modernization. Although population aging is a known driver of prostate cancer burden, the use of SIR in this study suggests that demographic structure alone does not fully explain these geographic variations [35, 36]. The persistent high risk in the southeastern coastal zones parallels the “Westernization” of behavioral patterns following rapid economic growth. Literature suggests this shift is characterized by increased consumption of red meat and high-fat foods, alongside reduced physical activity [34], both of which are established risk factors. Furthermore, the elevated detection rates in these economically developed regions may reflect greater accessibility to medical resources and opportunistic PSA screening. In contrast, the central and northwestern regions, characterized by the relative preservation of traditional lifestyles and lower screening intensity, exhibit a markedly lower risk profile [33].

While the current analysis did not incorporate ecological covariates to quantify their specific impacts, the spatiotemporal patterns observed herein warrant discussion regarding potential environmental and socioeconomic determinants. The high-risk clusters identified in this study coincide geographically with regions known for high industrial enterprise density and GDP levels [9]. Existing theoretical frameworks suggest that such urbanization and economic development may contribute to regional cancer variation beyond individual susceptibility. For example, prior studies have indicated that high population density may elevate community-level exposure to potential risk environments [37], and industrialization-related air pollution (e.g., elevated PM₂.₅) has been linked to increased regional burden in other analyses [38]. Additionally, disparities in healthcare resources likely influence these detection patterns: the southeast possesses abundant medical resources and higher population-level PSA screening rates [5, 39], which may enhance case identification at the regional level compared to the central and western regions. Future studies utilizing ecological regression models are needed to empirically test these associations.

This study further reveals three key spatiotemporal characteristics of prostate cancer distribution. It is important to emphasize that our inferences pertain strictly to area-level contexts and should not be extrapolated to individual-level conclusions (ecological fallacy). First, the observed variability is driven primarily by unstructured spatial effects, suggesting that area-level risk fluctuations stem mostly from independent random variation or localized noise rather than from broad regional clustering. Reviewing the potential sources of this unstructured variability, prior literature implies that transient factors, such as short-term environmental fluctuations [9, 40] or temporary shifts in localized screening efforts, which could contribute to such noise. However, as specific covariates were not modeled, these attributions remain hypothetical. Second, most regions exhibit negative spatiotemporal interactions, indicating a pattern where area-level risk increased less over time than would be expected from the separate spatial and temporal trends. This pattern is consistent with a scenario where certain regions may have experienced stabilizing effects, potentially from public health interventions as observed in other settings [41–43]. Third, the few regions exhibiting positive spatiotemporal interactions highlight areas where observed risk trends diverge significantly from the national pattern, potentially reflecting unmeasured contexts of cumulative environmental stressors or gaps in healthcare access [44].

These findings have significant implications for public health policy and practice. The identification of stable high-risk hotspots in the southeast suggests that resource allocation should be prioritized in these regions to manage the growing burden concentrated in specific geographic areas. Given that a significant portion of the risk variation is unstructured (localized), strategies should move beyond generalized national policies to targeted local interventions. Furthermore, the observation of negative spatiotemporal interactions in most regions implies that current public health contexts in these areas may be exerting a stabilizing effect. Conversely, in regions exhibiting synergistic risk interactions, the observed amplification likely reflects unmeasured contextual variables. Future inquiry should isolate these local deficits, such as healthcare access barriers for migrant populations or environmental exposures that the current model identifies as significant yet fails to specify.

Key limitations of this study must be acknowledged. First, regarding the study design, this is a descriptive ecological analysis intended for hypothesis generation rather than hypothesis testing. The study focused on identifying spatial patterns and clustering rather than quantifying statistical association through ecological regression. Therefore, the aggregate distributions observed at the area level do not necessarily reflect individual-level clinical risks, and causal inferences cannot be drawn from these descriptive data. Second, although we applied indirect standardization to calculate Standardized Incidence Ratios (SIRs) to adjust for the confounding effect of age, a limitation arises from the estimation of population denominators. Due to the unavailability of detailed age-specific population data at the individual city level, we estimated the age structure of each city based on the demographic composition of its corresponding province. This approach assumes that a city’s age structure mirrors its provincial average. Consequently, it may introduce measurement bias for specific cities where the demographic profile deviates significantly from the provincial norm (e.g., major economic hubs with large inflows of younger migrant workers). However, given the data constraints, this indirect standardization strategy represents a substantial methodological improvement over the use of crude rates, allowing for more valid spatial comparisons. Third, a significant limitation is the absence of area-level covariates (e.g., socioeconomic deprivation, healthcare accessibility, environmental exposure data) in Bayesian models. Incorporating such covariates is essential for explaining the drivers of spatial inequalities. Due to data limitations, we could not replicate this explanatory approach. Therefore, the discussion regarding potential drivers relies on theoretical frameworks and should be interpreted with caution. Finally, the temporal span (2013–2016) is relatively short, and the use of estimated city-level data from registry samples may introduce measurement error, particularly in central and western regions with lower registry coverage. Future studies should aim to integrate longer-term datasets, precise local census-based age structures, and comprehensive ecological covariates to corroborate the spatiotemporal patterns identified here.

## Conclusion

In conclusion, the study provides a comprehensive picture of the dynamic landscape of prostate cancer in China, revealing a persistent spatial gradient “high in the southeast and low in the northwest”, alongside a nationwide temporal upward trend. The findings indicate that unstructured spatial effects and negative spatiotemporal interactions are predominant, characterized by significant spatiotemporal clustering. This suggests that regional risk dynamics are complex, non-additive, and spatiotemporally dependent. These patterns reflect the interplay between underlying spatial determinants and stochastic yet geographically correlated local fluctuations, rather than simple linear trends. The study makes an important contribution to the literature by demonstrating the effectiveness of Bayesian spatiotemporal models in capturing disease dynamics in China. Future research should focus on addressing unresolved questions identified in this study, particularly by integrating individual-level data to validate these ecological associations and by exploring the specific causal mechanisms driving positive interactions in identified high-risk outlier areas.

## Supplementary Information

Supplementary Material 1.

## Data Availability

The data included in this study are available from the Annual Reports on Cancer Registration in China.

## Abbreviations

BYM: Besag-York-Mollié model
AR: Autoregressive model
PC: Penalized Complexity
DIC: Deviance information criterion
WAIC: Watanabe-Akaike information criterion
MoranST: Spatiotemporal Global Moran’s I
