# Supplementary material for: Spatiotemporal patterns and clustering of prostate cancer incidence in China: a Bayesian modeling study of cancer registry data
Source: BMC Public Health. 2026 Feb 14;26:973. doi: 10.1186/s12889-026-26619-7 (PMC13015175; doi:10.1186/s12889-026-26619-7)

**Supplementary Text 1: Detailed Model Selection Process**

Pure Spatial Models: We first compared the fit of pure Bayesian spatial models (Supplementary Table 2). The difference in DIC and WAIC values between the standard BYM model (Model 1) and the BYM2 model (Model 2) was negligible (DIC: 5026.56 vs. 5026.89; WAIC: 5050.72 vs. 5051.83), suggesting comparable overall goodness-of-fit. However, Model 2 was preferred due to its superior parameter interpretability. Unlike Model 1, where the non-independence of hyperparameters (τᵤ and τᵥ) complicates the separation of spatial components, Model 2 explicitly parameterizes the spatial structure using a mixing parameter (ω). In Model 2, the posterior mean of ω was 0.089, indicating that only 8.9% of the total spatial variance is attributable to structured spatial correlation, with the vast majority driven by unstructured variation (random noise).

Pure Temporal Models: For the temporal models (Supplementary Table 3), Models 3 and 8 yielded the best fit metrics. Although both models showed similar performance, Model 8 (using a Penalized Complexity [PC] prior) was selected as the optimal temporal model. The PC prior inherently favors parsimony, effectively penalizing complexity to prevent overfitting—a crucial advantage given that the temporal autocorrelation coefficients in both models were not statistically significant (P > 0.05).

Spatiotemporal Interaction Models: Among the 12 candidate spatiotemporal models, Model 12 (incorporating PC priors) provided narrower 95% CIs and enhanced interpretability, leading to its selection as the final optimal model. Sensitivity analysis confirmed the robustness of Model 12, as variations in hyperparameters resulted in negligible changes to DIC/WAIC (Supplementary Table 4). Ultimately, this selection process demonstrates that a model integrating temporal, spatial, and their interaction effects best captures the complex dynamics of prostate cancer incidence.

Supplementary Table 1. The global spatial and spatiotemporal autocorrelation coefficient of prostate cancer incidence from 2013 to 2016

| Year | Moran’s *I* | E (*I*) | Var (*I*) | Z value | P value |
| --- | --- | --- | --- | --- | --- |
| 2013 | 0.140532 | -0.002710 | 0.000059 | 18.625515 | <0.0001 |
| 2014 | 0.133355 | -0.002710 | 0.000059 | 17.678535 | <0.0001 |
| 2015 | 0.145188 | -0.002710 | 0.00006 | 19.171037 | <0.0001 |
| 2016 | 0.172540 | -0.002710 | 0.000059 | 22.730229 | <0.0001 |
| 2013-2016 | 0.6332 | -0.0019 | 0.000396 | 31.933 | <0.001 |

Supplementary Table 2. Results of simple Bayesian spatial models with different priors

| Models | Spatial effect | Spatial effect hyperparameter | Prior distribution | Parameter posterior estimation | | | DIC | WAIC |
| --- | --- | --- | --- | --- | --- | --- | --- | --- |
|  |  |  |  | Posterior mean | 95%CI | SD |  |  |
| Model 1 | BYM | *τu* | loggamma (1, 0.0005) | 2293.540 | (200.670, 8091.870) | 2191.521 | 5026.56 | 5050.72 |
|  |  | *τv* | loggamma (1, 0.0005) | 5.89 | (4.290, 5.810) | 0.922 |  |  |
| Model 2 | BYM2 | *τb* | pc.prec (1, 0.01) | 5.727 | (4.154, 7.727) | 0.910 | 5026.89 | 5051.83 |
|  |  | *ω* | pc (0.5, 0.5) | 0.089 | (0.002, 0.374) | 0.099 |  |  |

Supplementary Table 3. Results of simple Bayesian time models with different priors

| Models | Time effect | Time effect hyperparameter | Prior distribution | Parameter posterior estimation | | | DIC | WAIC |
| --- | --- | --- | --- | --- | --- | --- | --- | --- |
|  |  |  |  | Posterior mean | 95%CI | SD |  |  |
| Model 3 | RW1+iid | *τγ* | loggamma (1, 0.0005) | 22234.33 | (1551.26, 85392.48) | 23800 | 5309.32 | 5312.18 |
|  |  | *τϕ* | loggamma (1, 0.0005) | 22215.08 | (1444.95, 88917.01) | 25200 |  |  |
| Model 4 | RW1+iid | *τγ* | pc.prec (1.9, 0.09) | 997.74 | (2.45, 6927.39) | 6951.015 | 5311.11 | 5317.68 |
|  |  | *τϕ* | pc.prec (1.9, 0.09) | 1123.91 | (5.05, 7784.06) | 6469.719 |  |  |
| Model 5 | RW2+iid | *τγ* | loggamma (1, 0.0005) | 21469.55 | (1437.71, 80389.66) | 22200 | 5311.03 | 5316.81 |
|  |  | *τϕ* | loggamma (1, 0.0005) | 22379.94 | (1290.19, 95351.46) | 27800 |  |  |
| Model 6 | RW2+iid | *τγ* | pc.prec (2, 0.1) | 183.56 | (0.195, 1269.08) | 1571.125 | 5311.64 | 5318.78 |
|  |  | *τϕ* | pc.prec (2, 0.1) | 792.26 | (0.512, 5411.76) | 7884.170 |  |  |
| Model 7 | AR(1)+ iid | *τγ* | loggamma (1, 0.0005) | 2410 | (191.909, 9400) | 2640 | 5309.54 | 5313.14 |
|  |  | *ρ1* | normal (0, 0.15) | 0.055 | (-0.965, 0.091) | 0.654 |  |  |
|  |  | *τϕ* | loggamma (1, 0.0005) | 21800 | (1495.456, 14800) | 2220 |  |  |
| Model 8 | AR(1)+ iid | *τγ* | pc.prec (0.5, 0.07) | 148.628 | (82.281, 242.484) | 40.187 | 5310.70 | 5316.12 |
|  |  | *ρ1* | pc.cor0 (0.2, 0.2) | 0.008 | (-0.085, 0.103) | 0.048 |  |  |
|  |  | *τϕ* | pc.prec (0.5, 0.07) | 248.193 | (107.054, 435.605) | 85.309 |  |  |
| Model 9 | AR(2)+ iid | *τγ* | pc.prec (4, 0.05) | 93.942 | (16.451, 275.338) | 69.610 | 5310.34 | 5314.98 |
|  |  | *ρ1* | pc.cor0 (0.3, 0.5) | -0.384 | (-0.624, -0.094) | 0.136 |  |  |
|  |  | *ρ2* | pc.cor0 (0.3, 0.2) | -0.011 | (-0.122, 0.103) | 0.057 |  |  |
|  |  | *τϕ* | loggamma (1, 0.0005) | 6427.746 | (3312.406, 10728.338) | 1902.186 |  |  |
| Model 10 | AR(2)+ iid | *τγ* | pc.prec (3, 0.01) | 83.882 | (24.830, 220.649) | 52.085 | 5310.90 | 5316.26 |
|  |  | *ρ1* | pc.cor0 (0.3, 0.01) | -0.002 | (-0.070, 0.067) | 0.035 |  |  |
|  |  | *ρ2* | pc.cor0 (0.3, 0.3) | 0.030 | (-0.103, 0.181) | 0.072 |  |  |
|  |  | *τϕ* | pc.prec (3, 0.01) | 78.389 | (34.023, 143.958) | 28.364 |  |  |

Supplementary Table 4. Sensitivity Analysis

| Models | Change Form | Adjusted PC prior | DIC | WAIC |
| --- | --- | --- | --- | --- |
| Original Model | No Change | No adjustments | 4277.04 | 4298.94 |
| Sensitivity Model 1 | τb | pc.prec(2, 0.01) | 4276.99 | 4298.98 |
| Sensitivity Model 2 | τb | pc.prec(1, 0.05) | 4276.55 | 4298.22 |
| Sensitivity Model 3 | ω | pc(0.7, 0.5) | 4277.04 | 4299.01 |
| Sensitivity Model 4 | ω | pc(0.3, 0.5) | 4277.16 | 4299.17 |
| Sensitivity Model 5 | ρ1 | pc.cor0(0.4, 0.2) | 4276.66 | 4298.25 |
| Sensitivity Model 6 | ρ1 | pc.cor0(0.1, 0.2) | 4276.86 | 4299.03 |
| Sensitivity Model 7 | τγ | pc.prec(1, 0.07) | 4276.82 | 4298.69 |
| Sensitivity Model 8 | τγ | pc.prec(0.5, 0.1) | 4276.58 | 4297.62 |
| Sensitivity Model 9 | *τϕ* | pc.prec(1, 0.07) | 4276.46 | 4297.62 |
| Sensitivity Model 10 | *τϕ* | pc.prec(0.5, 0.1) | 4276.90 | 4298.90 |
| Sensitivity Model 11 | τδ | pc.prec(2, 0.01) | 4277.09 | 4299.17 |
| Sensitivity Model 12 | τδ | pc.prec(1, 0.05) | 4276.90 | 4298.57 |

Supplementary Figures

Supplementary Figure 1. Distribution of SIR (/100,000 population) of prostate cancer in (A) 2013; (B) 2014; (C) 2015.


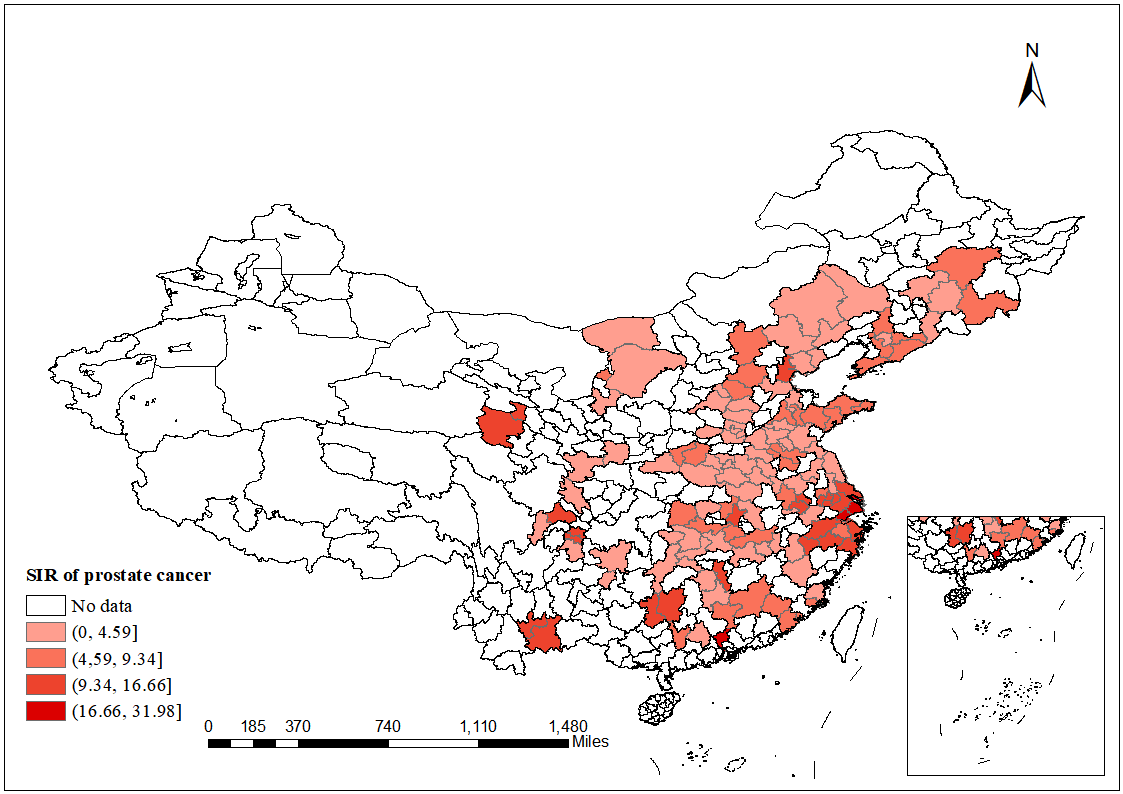


(A)


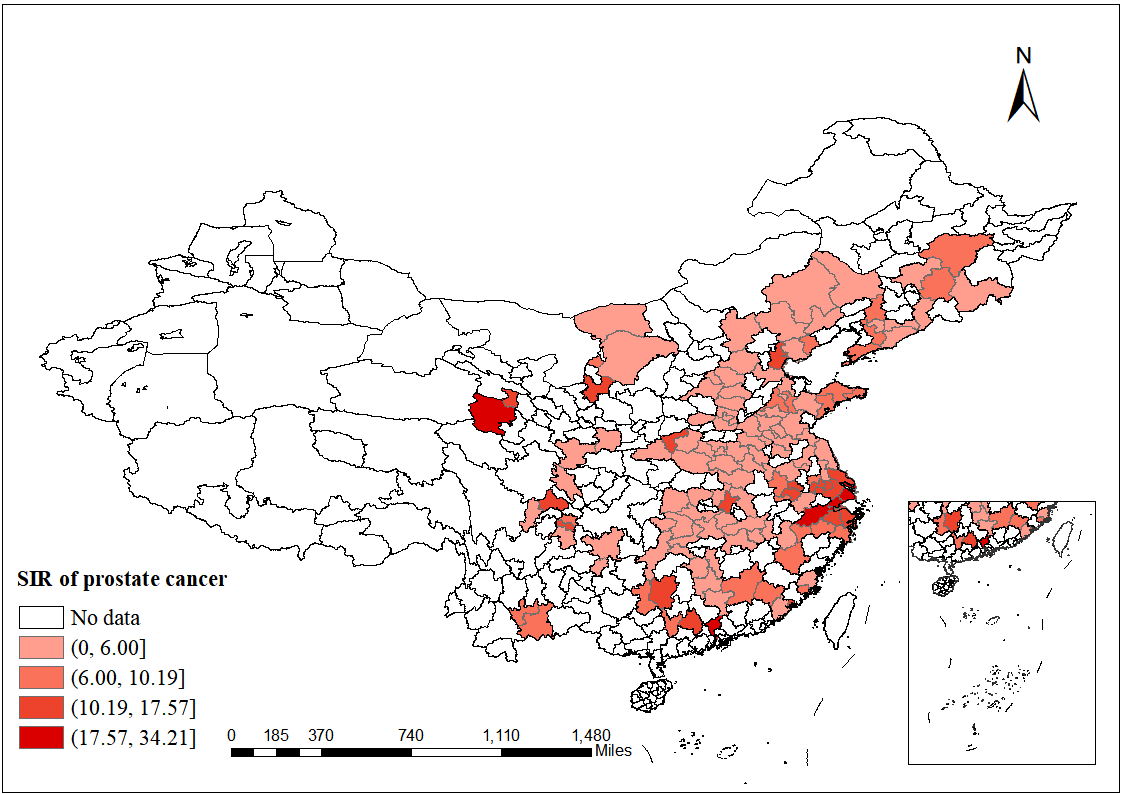


(B)


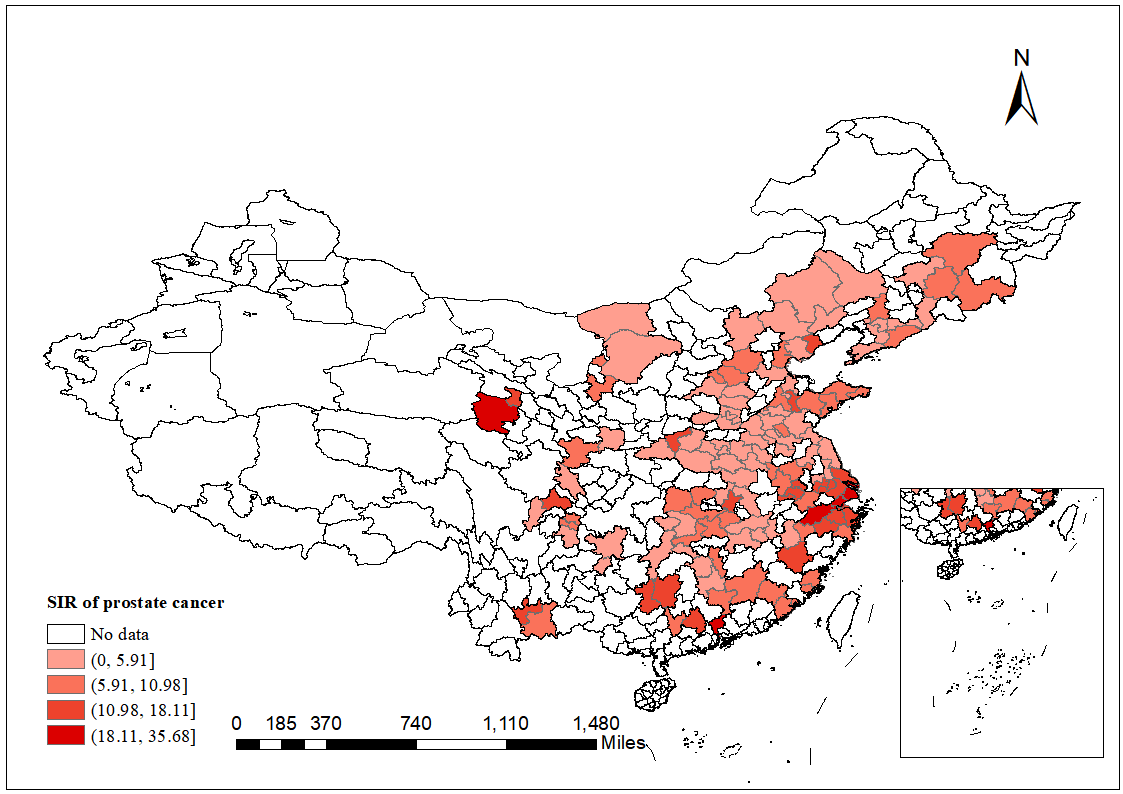


(C)

Supplementary Figure 2. The map of cluster and outlier analysis of prostate cancer in China in (A) 2013; (B) 2014; and (C) 2015.


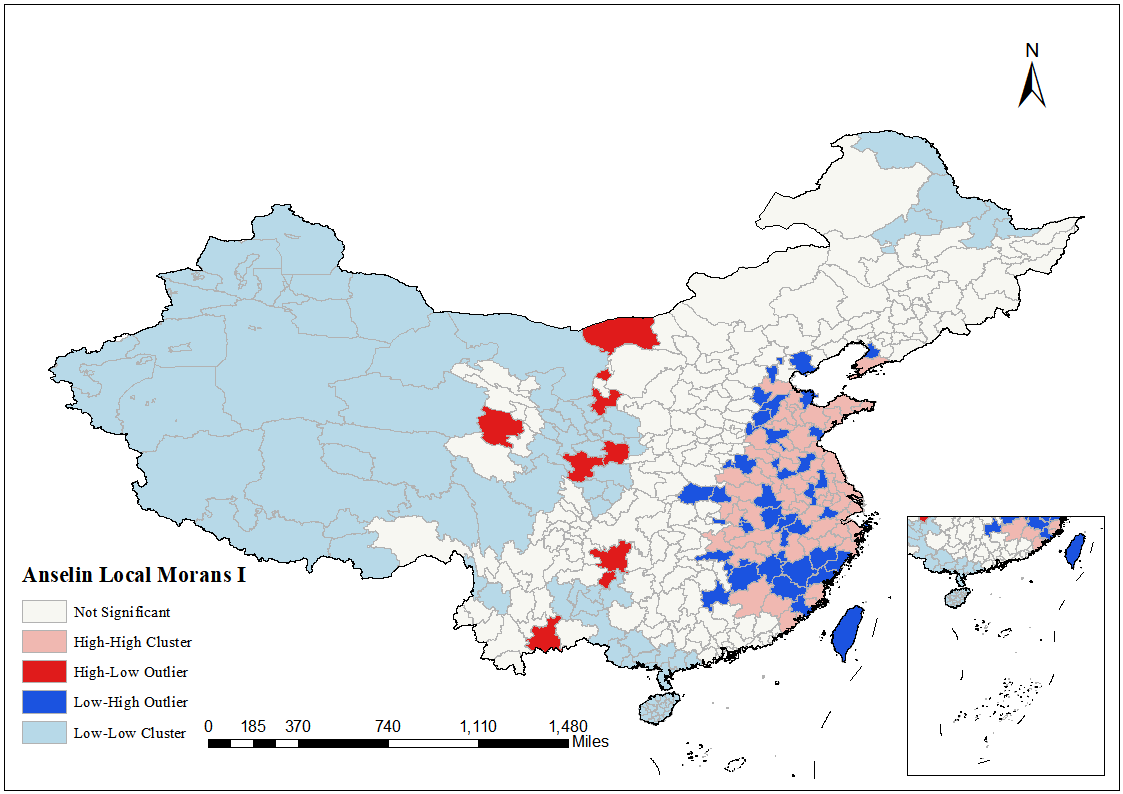


(A)
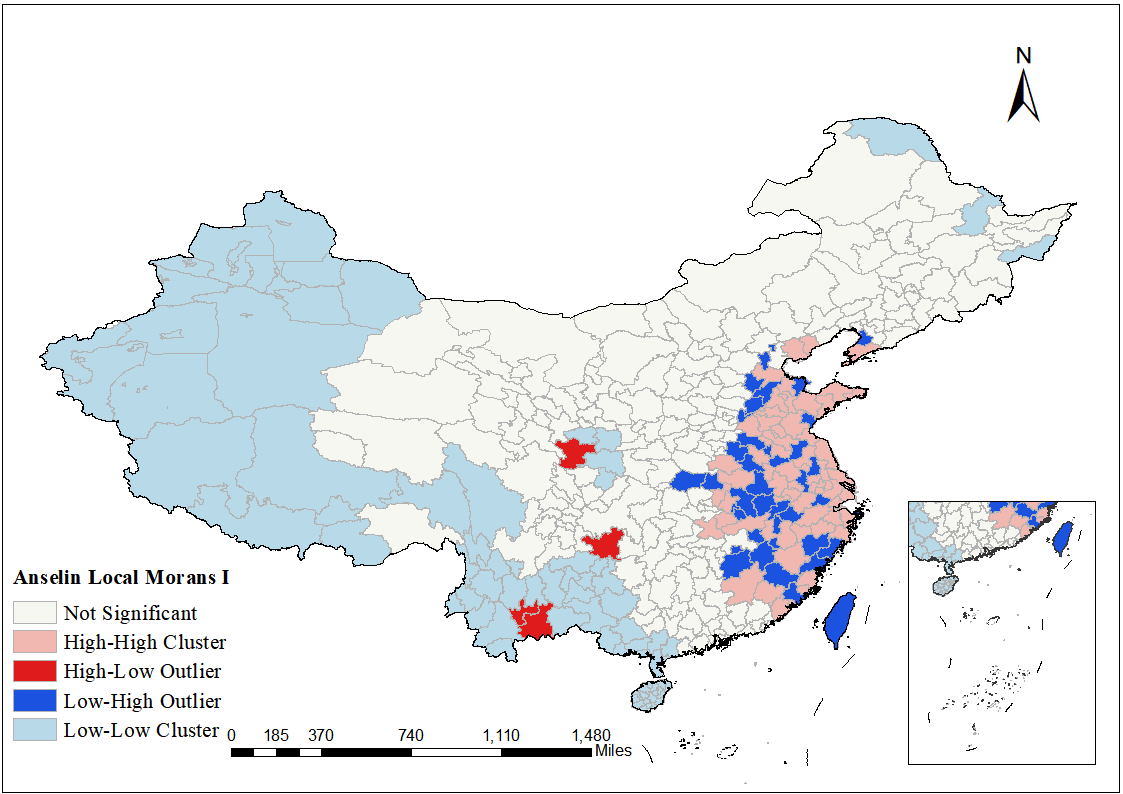


(B)
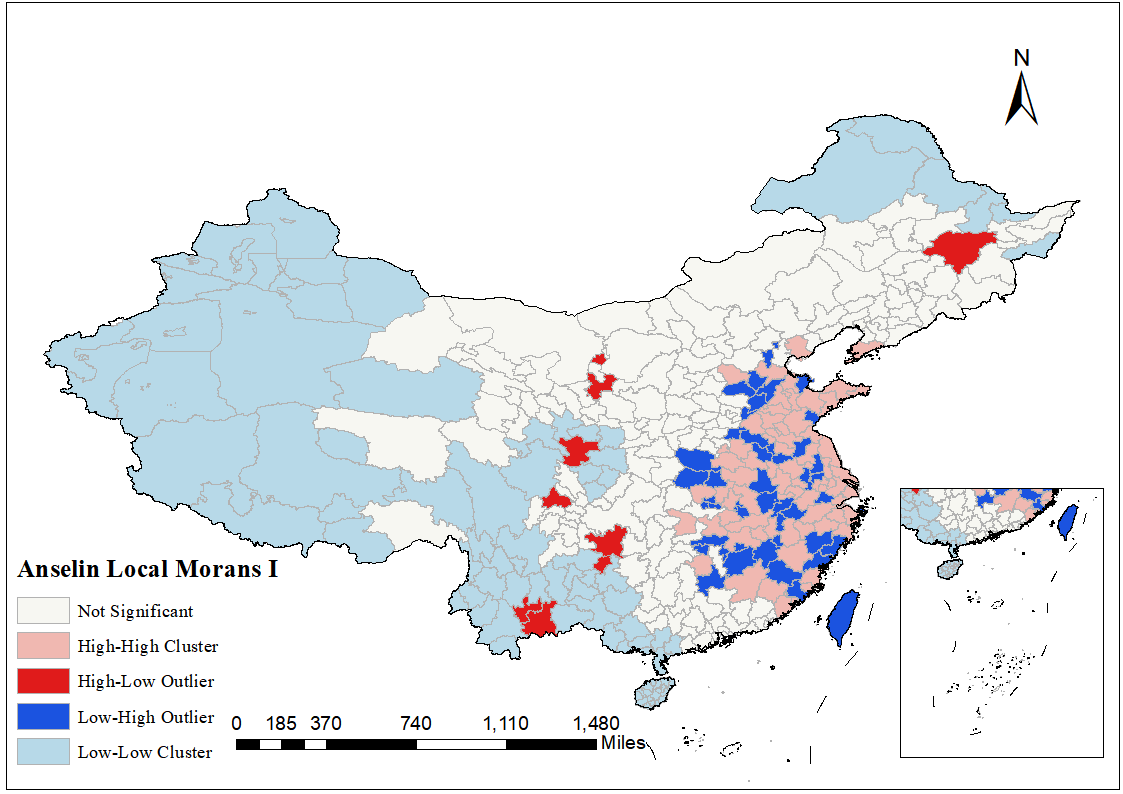


(C)

Supplementary Figure 3. The map of hot spot analysis of SIR of prostate cancer in China in (A) 2013; (B) 2014; and (C) 2015.


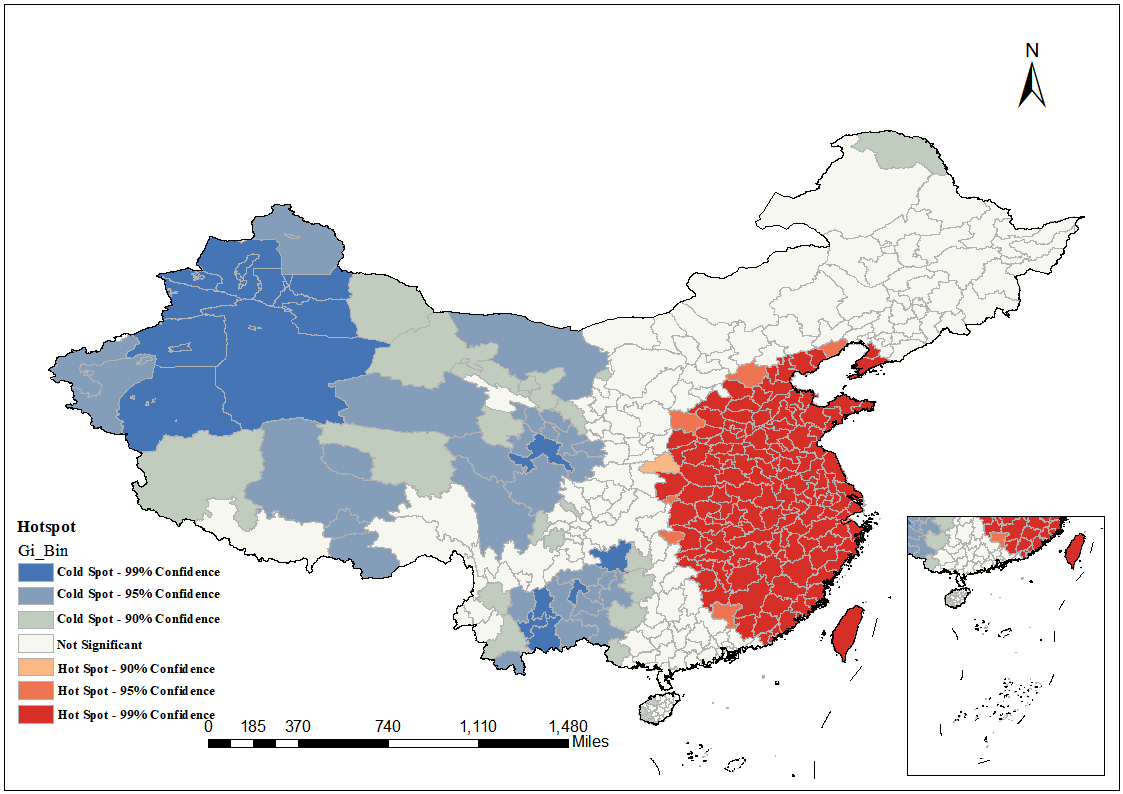


(A)
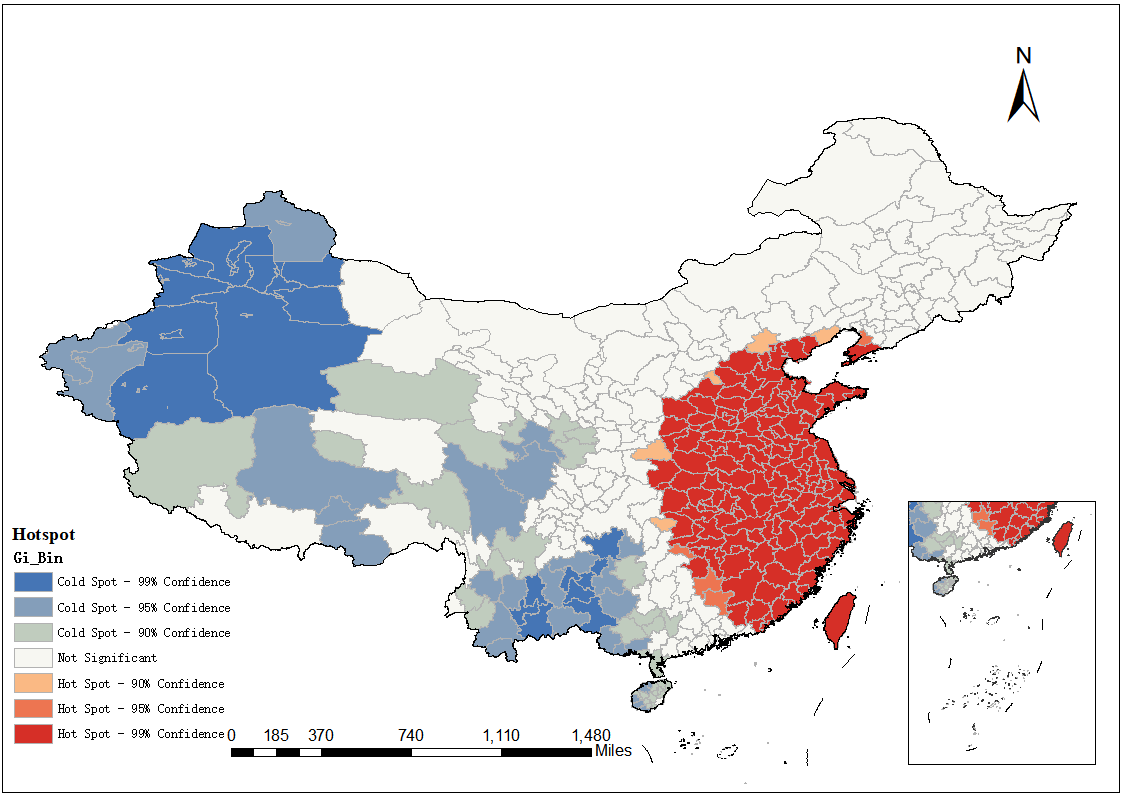


(B)
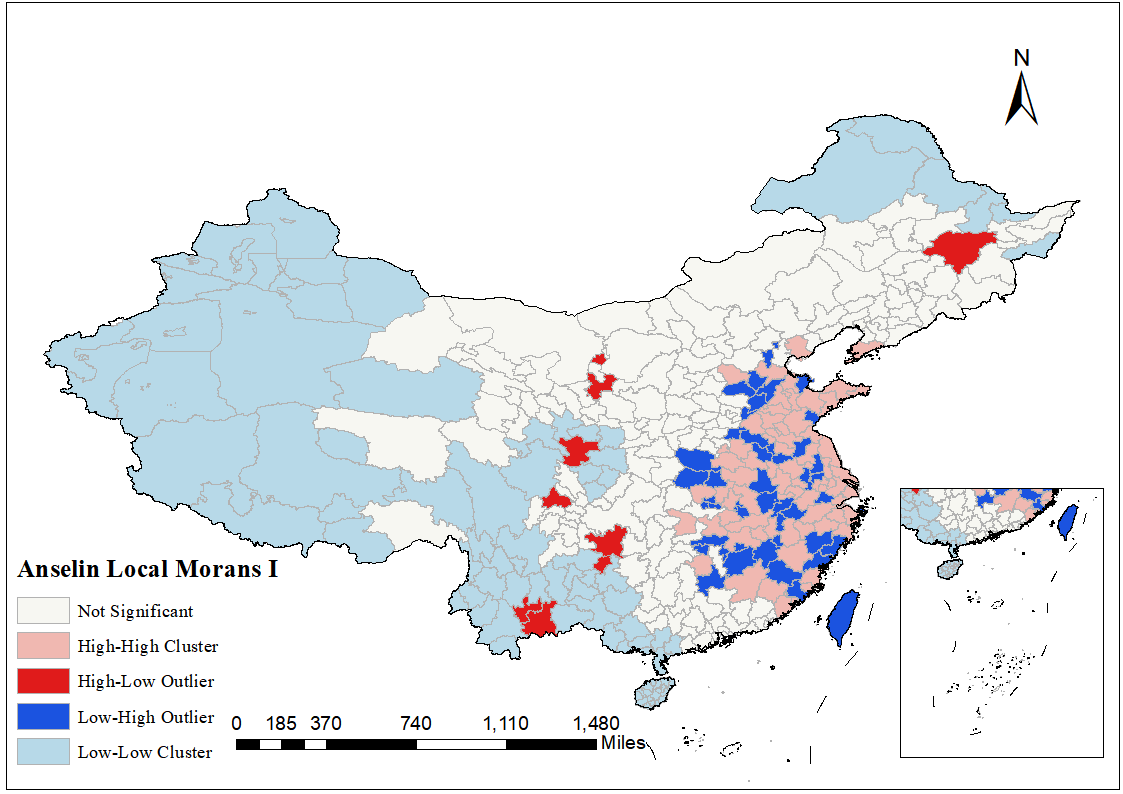


(C)

Supplementary Figure 4. PIT Histogram of the Model 12 (BYM2+AR(1)+ iid+ Besag (spatiotemporal interaction))


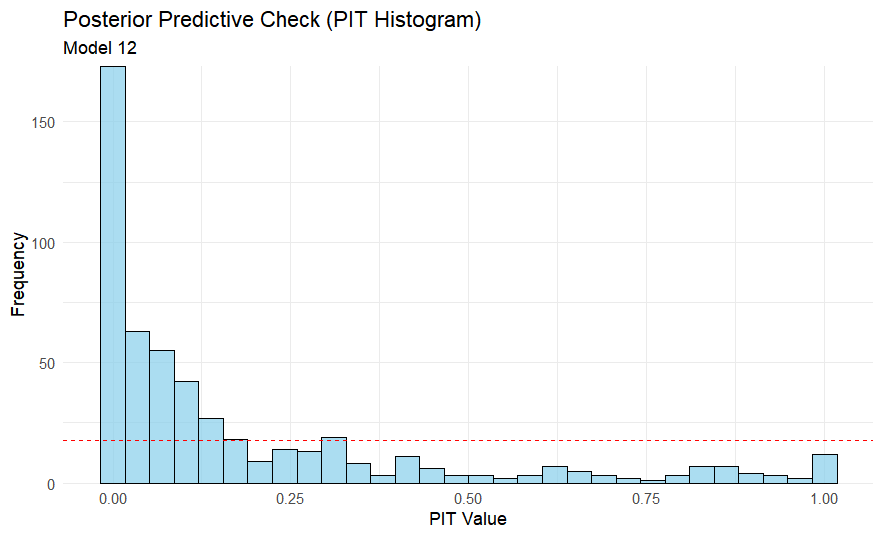

Supplement: Supplementary file 1 — Supplementary Material 1. [file 12889_2026_26619_MOESM1_ESM.docx]
